# Potential Roles of Glucagon-Like Peptide 1 Receptor Agonists (GLP-1 RAs) in Nondiabetic Populations

**DOI:** 10.1155/2022/6820377

**Published:** 2022-11-16

**Authors:** Dan Xu, Ankith Nair, Charlie Sigston, Chau Ho, Jia Li, Daya Yang, Xinxue Liao, Wei Chen, Ming Kuang, Yanbing Li, Christopher Reid, Haipeng Xiao

**Affiliations:** ^1^CCRE, Curtin School of Population Health, Faculty of Health Sciences, Curtin University, Australia; ^2^Curtin Medical School, Faculty of Health Sciences, Curtin University, Australia; ^3^Department of Medical Education, First Affiliated Hospital, Sun Yat-Sen University, Guangzhou, China; ^4^St John of God Midland Public and Private Hospitals, 1 Clayton St, Midland, WA 6056, Australia; ^5^Department of Pharmacy, First Affiliated Hospital, Sun Yat-Sen University, Guangzhou, China; ^6^Department of Cardiology, First Affiliated Hospital, Sun Yat-Sen University, Guangzhou, China; ^7^Department of Renal Medicine, First Affiliated Hospital, Sun Yat-Sen University, Guangzhou, China; ^8^Department of Surgery, First Affiliated Hospital, Sun Yat-Sen University, Guangzhou, China; ^9^Department of Endocrinology, First Affiliated Hospital, Sun Yat-Sen University, Guangzhou, China

## Abstract

Glucagon-like peptide 1 receptor agonists (GLP-1 RAs) have been observed in several large cardiovascular outcome trials to significantly reduce the incidence of major cardiovascular event (MACE) with type 2 diabetic patients. The clinical trials of GLP-1 RAs, including lixisenatide, exenatide, liraglutide, semaglutide, albiglutide, and dulaglutide, are associated with a significantly 14% lower risk of MACE in patients with T2DM and a history of CV disease, and with a nonsignificantly 6% lower risk in patients without history of CV disease. Some of the interpretation with GLP-1 RA trials suggested the possible role of glucagon-like peptide-1 receptor agonists (GLP-1 RAs) in primary prevention of cardiovascular diseases in nondiabetic individual, echoed by a recent editorial redefining the role of GLP-1 RAs being beyond glycaemic control. The narrative review provides an in-depth insight into GLP-1 RA use guideline in different countries and regions of the world and examines the safety and concern of GLP-1 RA use. The narrative review draws the comparison of GLP-1 RA use between diabetic and nondiabetic individual in terms of cardiovascular and metabolic benefits and points out the direction of future clinical trials of GLP-1 RAs in nondiabetic individuals. The focus of the review is on GLP-1 RAs' preventive roles in nondiabetic individuals with cardiovascular disease, chronic kidney diseases, obesity, dyslipidaemia, hypertension, nonalcoholic fatty liver diseases, polycystic ovarian syndrome (PCOS), and perioperative complications of bariatric surgery, albeit in small studies and subset analysis of clinical trials of diabetic patients.

## 1. Introduction

Glucagon-like peptide (GLP) was first discovered through research demonstrating a sustained insulin release in response to oral glucose load when compared with the intravenous glucose load [1]. GLPs' enhanced release of insulin in response to glucose ingestion became known as the ‘incretin effect' [2]. Further, study showed that patients with type 2 diabetes (T2D) had an impaired incretin effect after glucose load [3]. Subsequently, glucagon-like peptide 1 (GLP-1) was found to be specifically effective in stimulating insulin and reducing peak plasma glucose concentrations [4]. GLP-1 is released from the distal bowel within minutes of a meal and does enhance glucose-dependent insulin production and secretion, decrease glucagon secretion, increase glucose uptake and glycogen synthesis in peripheral tissues, delay gastric emptying, and increase satiety [5], making it an ideal target for diabetes therapy. The first GLP-1 receptor agonist (GLP-1 RA) was exenatide, which was approved by the US Food and Drug Administration (FDA) in 2005 for the treatment of T2DM [6]. Since then, several GLP-1 RAs have been recommended for treatment of diabetes as a second line therapy in view of their clinical efficacies including improved weight loss, low risk for hypoglycaemia, and reduction in glycated haemoglobin (HgA1c). GLP-1 RAs have its unique mechanism of action of simultaneously increasing insulin secretion and inhibiting glucagon only in response to increased glucose levels [7, 8] and can potentially be used in obese and nondiabetic individuals without the risk of hypoglycaemia [9]. In addition, GLP-1 RAs have some potential roles other than glycaemic control that was echoed by a recent editorial by Chowdhury and Goswami [10]. This study reviewed the roles of glucagon-like peptide 1 receptor agonists (GLP-1 RAs) beyond diabetes treatment based on available evidence in human studies.

## 2. Methods

The authors performed an extensive literature review in Ovid MEDLINE, Ovid Embase, CINAHL, Web of Science, and PsycINFO (Ovid) by using the following Medical Subject Heading (MeSH) terms including GLP-1 RA, guideline, safety, cardiovascular disease, chronic kidney diseases, obesity, dyslipidaemia, hypertension, nonalcoholic fatty liver diseases, polycystic ovarian syndrome, and perioperative complications of bariatric surgery. The review has identified 4594 publications related to GLP-1 RAs' safety, guidelines in different countries, and potential preventive roles. After scanning the titles and the abstracts to remove duplication, case reports, and editorial comments, we have included 75 publications for full text screening to identify the relevant discussion points regarding GLP-1 RA's potential roles in nondiabetic population in this review. The authors have applied the Oxford Equator PRISMA checklist [11] to satisfy an evidence-based narrative review. The Oxford Equator PRISMA checklist is an updated internationally-recognised guideline for reporting narrative and systemic reviews to ensure the transparency and integrity of the search source as well as the validity of the search methodology [11].

### 2.1. Current Recommendations for the Use of GLP-1 RAs in Clinical Practice

GLP-1 RAs have high efficacy on controlling blood glucose with low risk of hypoglycaemia like metformin. They have beneficial effects on CVD prevention, albuminuria, and weight loss similar to Sodium Glucose Cotransporter 2 (SGLT2) inhibitors [12]. GLP-1 RAs' guidelines of use in clinical practice varied in different countries, which is shown in Table 1. GLP-1 RAs can be indicated with or without metformin and are recommended in T2DM patients with CVD risk or CKD without albuminuria or those who need assistance for weight loss in the USA [12], in Australia [13], in European Union [14], in United Kingdom [15], and in China [16]. GLP-1 RAs are also recommended for obesity and overweight management in nondiabetic patients except in Chinese guideline [17]. Four common GLP-1 RAs that have been approved in Australia, the US, and in China are exenatide, dulaglutide, liraglutide, and semaglutide, which are administered subcutaneously except for oral semaglutide described in the US guideline [12]. The convenience of once-weekly semaglutide and dulaglutide may improve the adherence to treatment and reduce the burden of the daily administration as other hypoglycaemics.

### 2.2. GLP-1 RAs' Roles beyond Diabetes Treatment

Table 2 summarised the current evidence of GLP-1 RAs' role in both diabetic and nondiabetic patients.

### 2.3. Prevention of Cardiovascular Events in Both Diabetic and Nondiabetic Patients

GLP-1 RAs have been suggested in several major cardiovascular outcome trials (CVOTs) as potential treatment not only for T2DM and atherosclerotic cardiovascular disease but also in T2DM patients with manifest HF. In the LEADER trial in T2DM patients, liraglutide against placebo decreased the risks of cardiovascular death, myocardial infarction, and stroke with no significant effects on HF hospitalizations [18]; similar outcomes were also observed in the SUSTAIN-6 semaglutide against placebo control trial [19]. In contrast, the ELIXA [20], EXSCEL [21], REWIND [22], and the HARMONY Outcomes trial [23] showed no significant effect on a composite outcome of HF hospitalization and cardiovascular death. However, the HARMONY trial [24] did show lower risk of HF hospitalization with GLP-1 RAs versus placebo with a hazard ratio [HR] of 0.71 (95% CI). The PIONEER 6 trial [25] also found no difference between GLP-1 RAs and placebo in HF hospitalizations. A meta-analysis of seven trials [25] including >56 000 T2DM patients, GLP-1 RAs showed modest but statistically significant reductions in MACE by 12%, all-cause mortality by 12%, a broad kidney composite outcome by 17%, and a slightly lower risk of hospitalization for HF by 9%, in contrast to observations and conclusions from individual randomized trials suggesting no clear benefit on HF-related outcomes and even uncertainty regarding the safety in those with HF with reduced ejection fraction. In the subset of diabetic patients with baseline HF, the EXSCEL trial [26] recruiting T2DM patients with and without HF showed GLP-1 RA was not associated with a significant reduction of all-cause mortality, whereas the association was significant in the subgroup without HF. In the post hoc analyses of individual patient-level data combined from SUSTAIN-6 and PIONEER 6, semaglutide showed no difference on MACE in patients with baseline HF [27]. Similar results were observed in the post hoc analysis of LEADER trial with the treatment effect of liraglutide on MACE [28]. In patients of chronic HF with reduced Ejection Fraction (HFrEF), two liraglutide against placebo trials [29, 30] showed no differences in HF-related outcomes or functional capacity in HFrEF patients with and without diabetes.

Further exploration of GLP-1 RAs in nondiabetic patients, a mild improvement of left ventricular function has recently been demonstrated in patients with ST-segment elevation myocardial infarction (STEMI) treated acutely with the GLP-1 analogue liraglutide for 7 days [31]. Similar results were shown in patients with non-STEMI irrespective of diabetic status [32]. It raised the possible preventive role of GLP-1 RAs on cardiovascular events in nondiabetic population that needs to be confirmed in future studies. Taking together, there is no convincing trial evidence with GLP-1 RA for prevention of cardiovascular events in nondiabetic population.

### 2.4. Prevention of CKD in DM and Non-DM Patients

In placebo control trial with diabetic patients, GLP-1 RAs reduced the risk of the broad composite kidney outcome significantly by 17%, in particular with a 26% reduction in macroalbuminuria [33]. To date, there are no human trials studying renal outcomes of GLP-1 receptor agonists in nondiabetic patients.

The analysis of renal endpoints in large placebo-controlled CVOTs of GLP-1 RAs in patients with T2DM either with cardiovascular comorbidities or at high risk of cardiovascular disease suggests that GLP-1 RAs may be renoprotective even when corrected for glycaemic control [33]. GLP-1 RA is thought to inhibit sodium-hydrogen exchange in the proximal-convoluted tubules, thus increasing urinary sodium excretion [34]. In ELIXA, lixisenatide reduced urinary albumin-to-creatinine ratio; although correction for glycaemic control decreased significance, diminished the incidence of macroalbuminuria, remaining unattenuated when corrected for HbA1c, and did not affect eGFR decline [20]. In LEADER, liraglutide decreased poor renal outcomes by 22% and in SUSTAIN-6, semaglutide reduced poor renal outcomes by 36%, while post hoc analyses showed that their antialbuminuric effects were independent of their glucose-lowering effects. Both drugs also induced a slower rate of eGFR decline [18, 19]. In EXSCEL and REWIND, exenatide and dulaglutide, respectively, lowered the incidence of macroalbuminuria after adjusting for HbA1c, without affecting eGFR [21, 22]. HARMONY and PIONEER 6 reported no change in eGFR with albiglutide and semaglutide, respectively [23, 24]. GLP-1 RAs reduced blood pressure after prolonged treatment, specifically lowering systolic blood pressure by 3-4 mmHg, which is renoprotective. Similarly, weight loss in obese patients up to 2 kg associated with lowering blood pressure suggests GLP-1 RAs' benefit in patients with CKD even without T2DM [35]. No evidence on the effect of GLP-1 RAs on CKD has been reported. In summary, there is no direct trial evidence of GLP-1 RAs' effects on prevention of CKD in nondiabetic population (Table 2).

### 2.5. Prevention of Obesity/Overweight in Diabetic and Nondiabetic Patients

Obesity is on the rise and has now been defined as a chronic disease with serious health consequences and associated with increasing prevalence of type 2 diabetes [36, 37]. A systematic review [38] has summarised the evidence that weight loss of 5 to 10% reduce obesity-related complications and improve quality of life. Maintaining weight loss with lifestyle intervention alone has proven to be difficult [39].

While changes in diet, behavioural modifications, and physical activity have typically been the foundation of weight management, weight loss achieved by these interventions alone is often limited and unsatisfactory [40]. Bariatric surgery has been proven effective but carry associated surgical risks and typically used as a last resort [41].

Pharmacotherapy may be considered in obese patients with a BMI of 30 and above or in patients with lower BMIs with weight-related complications [40]. The development of GLP-1 RAs have created a new avenue for the management of obesity in patients with and without T2DM [40]. Current research with GLP-1 RAs has provided significant evidence, especially liraglutide accounting for up to 56% of the global pharmacological approach to obesity management. Long-acting analogues of GLP-1 RAs showed good weight loss activity [42]. Recent review has shown clear evidence of GLP-1 RAs to reduce body weight by 5.8-9.6% in obese adults with T2DM [43]. Liraglutide has been shown to result in weight loss that is dose-dependent, up to 3.0 mg daily [44]. A 56-week RCT of liraglutide 3.0 mg daily, used as an adjunct with a reduced calorie diet and increased physical activity in nondiabetic overweight or obese adults demonstrated a significant averaged weight reduction by −8.4 kg ± 7.3 kg [44], corresponded to an averaged reduced BMI of −3.0 ± 2.6 kg/m2 [44]. This study also found that liraglutide is associated with reduced cardiometabolic risk factors including waist circumference, blood pressure, and inflammatory markers [44].

Another RCT comparing semaglutide with liraglutide and placebo in nondiabetic patients demonstrated a 7.8% weight loss in the liraglutide 3.0 mg group [45]. Interestingly, however, the study found a higher average weight loss in the semaglutide group of 11-14% at doses of 0.2 mg daily or more [45]. The average weight loss was found to be dose-dependent with reduction of both fat mass and lean mass at a rate of 3 : 1 on 1.0 mg daily [45]. A meta-analysis of 574 children and adolescents with obesity but nondiabetic in 9 RCTs highlighted the impacts of GLP-1 RAs including liraglutide and exenatide in modestly reducing weight, BMI, glycated haemoglobin A1c, and systolic blood pressure [46].

In summary, GLP-1 RAs have been well placed as the forerunner of pharmacological intervention for obesity in both diabetic and nondiabetic population (Table 2).

### 2.6. Prevention of Dyslipidaemia and Hypertension

There are no direct clinical trial examining GLP-1 RA effects on cardiometabolic variables including blood pressure and dyslipidaemia. In the recent liraglutide trial with obese nondiabetic patients, both changes between baseline and week 56 of systolic and diastolic blood pressure were significantly different between the liraglutide group and the placebo group with a risk difference of -2.8 mmHg (95%Cl -3.56 to -2.09) and -0.9 (95%Cl -1.41 to -0.37), respectively [44]. ,Similarly, the reduction of total cholesterol from baseline to week 56 was significantly higher in the liraglutide group with a risk difference -2.3% (95%Cl -3.3 to -1.3) [44]. In summary, there is no direct trial evidence of GLP-1 RAs' effects on prevention of CKD in nondiabetic population (Table 2). A dedicated trial should be carefully designed to examine cardiometabolic endpoints including blood pressure, lipid, and other metabolic variables with GLP-1 RAs in nondiabetic population.

### 2.7. Prevention of Nonalcoholic Fatty Liver Disease

Nonalcoholic fatty liver disease (NAFLD) is highly prevalent in diabetic patients associated with ASCVD, diastolic dysfunction, and HFpEF. More than one-third of patients with NAFLD have diastolic dysfunction, coronary microvascular dysfunction, and myocardial fibrosis [47, 48]. In obese patients with nonalcoholic steatohepatitis (32% having diabetes), the LEAN trial using liraglutide or placebo in small group of patients showed more frequent resolution of NAFLD and less progression of fibrosis [49]. However, there was no subgroup analysis with the LEAN trial [49] for this effect of liraglutide in nondiabetic population. Another small study [50] randomly assigned diabetic patients to liraglutide, metformin, or gliclazide for 6 months, with patients in the liraglutide arm showing the greatest reduction in intrahepatic fat. Similar results have been reported with exenatide [51]. A meta-analysis including 6 studies showed that GLP-1 RAs reduce circulating ALT levels and improve NAFLD [52]. Another meta-analysis found significant improvements in hepatic fat content, liver biochemistry, body composition, glucose parameters, lipid parameters, insulin sensitivity, and inflammatory markers following GLP-1 RA treatment [53].

In summary, there is no direct trial evidence of GLP-1 RAs' effects of improving and preventing NAFLD in nondiabetic population.

### 2.8. Prevention of PCOS in Nondiabetic Patients

GLP-1 RAs have been studied in the treatment of PCOS because of their effectiveness in reducing weight and the associated improvement in hyperandrogenism and metabolic parameters [54]. GLP-1 RAs have also provided beneficial effects on insulin sensitivity, thus addressing many manifestations of PCOS as approximately 70% of women are insulin resistant and 80% are overweight or obese [54]. GLP-1 RAs demonstrated significant improvements in fasting blood glucose, triglycerides, total cholesterol, and homeostasis model assessment for insulin resistance in a systematic review [54]. This trial featured by exenatide and metformin effects on the remission of prediabetes, a prevalent comorbidity of PCOS [54]. The trial of 150 women with PCOS with impaired fasting glucose and/or impaired glucose tolerance demonstrated a prediabetes remission rate of 64% with combined treatment, 56% with exenatide only and 32% with metformin only [54]. Similarly, a meta-analysis of six randomised-controlled trials concluded that GLP-1 RAs were significantly associated with lower BMI and HOMA-IR when compared to metformin [55].

Open-label clinical trials exploring the effect of exenatide on body mass in overweight or obese women with PCOS showed a significant reduction in body weight, ranging from -3.1 to -6 kg and a corresponding decrease in BMI between -1 and -3.1 kg/m^2^ [54]. In addition, a meta-analysis of RCTs also identified a significant improvement in reducing BMI and abdominal girth of women with PCOS, when compared to metformin [56].

The systematic review examined the effects of GLP-1 RAs on PCOS-related subfertility and irregular periods. Exenatide compared with metformin demonstrated a significantly greater number of spontaneous pregnancies in the exenatide group, after 24 weeks of treatment [54]. Another 12-week trial demonstrated a significantly increased frequency and regularity of menstrual cycles with exenatide [54]. Similar findings were demonstrated in a trial of overweight, oligogovulatory women with PCOS, with an increase in the regularity of menses and a higher rate of ovulation in the single-agent exenatide and metformin groups [54]. Interestingly, the combination of exenatide and metformin resulted in a higher rate of ovulation and menstruation than the single-agent groups in this trial [54].

In summary, there is some trial evidence of GLP-1 RAs' effects on prevention of PCOS through weight reduction in nondiabetic population (Table 2).

### 2.9. Potential Roles of GLP-1 RA on Prevention of Perioperative and Postoperative Complications of Bariatric Surgical Individual

Bariatric surgery is the most commonly performed procedures and the most effective intervention for obesity with BMI over 30 kg/m^2^ [57]. GLP-1 rises sharply after bariatric surgery, accelerating nutrient transit to the distal gastrointestinal tract and increases secretion of incretin and satiety peptides with tenfold higher postprandial GLP-1 levels [58]. GLP-1 is proposed to decrease appetite, decrease fasting insulin, increase postprandial insulin, and decrease hepatic glucose production, even in patients without T2DM after bariatric surgery. GLP-1 receptors in the CNS are also thought to regulate body weight; in rats, GLP-1 antagonists infused into the CNS induced weight gain [59]. A recent randomised, double-blind, placebo-controlled clinical trial [60] *“*GLP*-*1 Receptor Agonist interVentIon for poor responders afTer bariAtric Surgery (GRAVITAS)” investigated the safety and efficacy of subcutaneous injections of the liraglutide on glycaemic control in patients with persistent or recurrent T2DM after bariatric Roux-en-Y gastric bypass or vertical sleeve gastrectomy. The conclusion drawn from the study stated that irrespective of the type of surgery, liraglutide can treat the persistent or recurrent postbariatric surgery complication of T2DM by optimising glycaemic control along with a needful weight loss. The GRAVITAS trial raised the questions of GLP-1 RA's role especially preoperatively to aid on reducing perioperative complication beyond diabetes and other associated cardiovascular complications in nondiabetic patients as there is currently no direct trial evidence of GLP-1 RAs' effects on prevention of perioperative and postoperative complications of bariatric surgical individual, especially in nondiabetic population (Table 2). Further, properly-designed clinical trials may answer these questions in nondiabetic bariatric-surgical patients for the prevention of perioperative complications and consolidation of GLP-1 RA's weight loss benefit postbariatric surgery.

### 2.10. GLP-1 RAs Safety and Adverse Events

There are numerous reports and reviews of potential safety issues associated with the use of GLP-1 RAs including gastrointestinal symptoms, hypoglycaemia, pancreatitis, injection site reaction, and allergy/angioedema.

#### 2.10.1. Gastrointestinal Symptoms

The most frequently reported adverse effects of GLP-1 RA therapy were gastrointestinal symptoms [61]. Of these symptoms, nausea was the most common and earliest complaint, occurring in up to 50% of patients [62, 63], and others included vomiting, diarrhea, constipation, dyspepsia, and abdominal pain [64].

Second commonly reported adverse effect of GLP-1 RA was gastroparesis. GLP-1 RA have been shown to delay gastric emptying that may increase the risk of nausea, and longer duration of action may have a lesser effect on gastric motility, thus leading to less nausea [65]. Long-acting GLP-1 RAs such as weekly regimen or XR form was less likely to associate with nausea as compared to short-acting and frequent daily regimen [66, 67]. In another study, gastric half-time emptying was delayed in most diabetic patients without preexisting gastroparesis after commencing GLP-1 RAs, while those with preexisting gastroparesis were minimally affected [68].

### 2.11. Hypoglycaemia

Generally, the risk of hypoglycaemia in patients taking GLP-1 RAs alone was low, and similar across all GLP-1 RAs [66, 69]. The combination of GLP-1 RAs and metformin has not been associated with increased rate or severity of clinically relevant hypoglycaemic events [70, 71]. In clinical trials examining the use of GLP -1 RAs and sulfonylureas, the incidence of hypoglycaemia was increased, when compared to the placebo group [72]. This hypoglycaemic effect has been attributed to the effect of SU uncoupling the action of GLP-1 RA from its glucose dependence. GLP-1 RAs do not treat hypoglycaemia; however, they may be used as an alternative to other antidiabetic drugs that do pose a risk of hypoglycaemia, as GLP-1 RAs do not generally cause hypoglycaemia [72].

#### 2.11.1. Pancreatitis, Pancreatic Hyperplasia, and Neoplasia

There has been conflicting evidence on GLP-1 RAs use and risks of pancreatitis including some studies suggesting a slightly increased risks [73], while others evidence showed no significant risks [74, 75].

Overall, there has not been a clear cause and effect relationship described between GLP-1 RAs and pancreatitis [76, 77]. There is no long-term safety data for the administration of GLP-1 RAs in nondiabetic patients; however, all major CVOTs in diabetic patients support the safety of GLP-1 RAs in the long-term, with no increase in severe hypoglycaemia, pancreatitis, or pancreatic neoplasms [78].

#### 2.11.2. Injection Site Side Effects

Symptoms that may occur include pruritis, rash, or erythema, with pruritis being the most frequently reported injection site symptom [72]. Generally, these symptoms are common and transient in nature. Additionally, injection site reactions are reported more frequently with long acting than short-acting GLP-1 RAs [67]. In phase II and III trials, these reactions occurred in 5.1% of patients receiving exenatide twice daily and 16% in patients receiving exenatide once weekly [72]. In patients receiving lixisenatide, 3.9% of patients experienced injection site reactions [72].

#### 2.11.3. Allergy/Angioedema

GLP-1 RAs are synthetic peptides, which may lead to antibody formation, like other subcutaneously administered peptides [72]. Antibody formation was seen in 69.8% of patients receiving lixisenatide, 44% with exenatide, 8.6% with liraglutide, and 1.6% with dualglutide [72]. Adverse event rates were similar between patients who were antibody positive and antibody negative, with the exception of injection site reactions; these occurred more commonly in patients who were antibody positive [72]. Additionally, a significant decrease in the efficacy of exenatide was noted in patients with high antibody titres [72]. Severe anaphylactic reactions are rare in patients receiving GLP-1 RAs; however, rare postmarketing reports of urticaria, pruritis, and angioneurotic edema were made with exenatide, lixisenatide, and liraglutide [72].

## 3. Conclusion

As summarised in Table 2, GLP-1 RAs showed modest but statistically significant reductions in MACE, all-cause mortality, a broad kidney composite outcome of reduced macroalbuminuria, and a slightly lower risk of hospitalization for HF in diabetic population, while observations and conclusions from randomized trials suggested no clear benefit on HF-related outcomes. However, GLP-1 RAs have only very limited and suggested evidence in terms of cardiovascular and renal outcomes in nondiabetic population. Furthermore, GLP-1 RAs have only been shown in several small studies and subset analysis of clinical trials in both diabetic and nondiabetic population to contribute to the management of blood pressure lowering, lipid reduction, prevention of PCOS complications, and contribution of assisting weight management in bariatric surgical patients. The gaps with the majority of these evidence lie in the fact of most studied population being diabetic patients or obesity patients with only small numbers of low-risk/non-CVD individuals. Taking all the evidence together, there is a need for consideration of GLP-1 RA as a potential agent for primary prevention clinical trial in low risk of cardiovascular, renal diseases, overweight, or even healthy population with research design focusing on both cardiovascular outcomes and cardiovascular risk reduction. A carefully-designed protocol for this GLP-1 RA double-blind randomised-controlled primary prevention trial should address the ultimate goal of exploring its effect on nondiabetic and non-CVD population in preventing cardiovascular disease, chronic kidney disease, obesity, dyslipidaemia, hypertension, NAFLD, PCOS, and perioperative complications of bariatric surgery.

## Figures and Tables

**Table 1:** Summary of GLP-1 RA guidelines in different countries.

	Australian guideline	The US guideline	Chinese guideline	EU guideline	UK guideline
Approved GLP-1 RA use	Exenatide Dulaglutide Liraglutide Semaglutide	Exenatide Dulaglutide Liraglutide Semaglutide Lixisenatide	Exenatide Dulaglutide Liraglutide Semaglutide Loxenatide Benaglutide	Exenatide Dulaglutide Liraglutide Semaglutide	Exenatide Lixisenatide Dulaglutide Liraglutide Semaglutide

Recommendations	Second line agent after metformin in T2DM patients				Fourth line as add on to metformin and SU therapy if BMI > 35 or significant issues with insulin use
	First line in T2DM with CVD risk, CKD without albuminuria Obesity/overweight without diabetes		First line in T2DM with CVD risk, CKD without albuminuria, and obesity/overweight Not for obesity and overweight without diabetes	First line agent in T2DM with established or high risk of CVD	

Concerns	GI side effects are common, increased risk of hypoglycaemia with metformin or SU therapy, pancreatitis, and gall bladder disease	GI side effects are common, injection site reactions, pancreatitis, FDA black box—risk of thyroid C-cell tumours in rodents, and human relevance not determined	Contraindicated for use in pregnant or lactating women, patients with severe gastrointestinal disease, a history or high risk of pancreatitis, and not for eGFR < 30 ml/min	GI side effects are common, less effective than SGLT2i at reducing HF hospitalisations	GI side effects are common, not cost effective as first line therapy

SU: sulphonylurea.

**Table 2:** Summary of GLP-1 RA preventive effects in DM and non-DM patients.

	DM	Non-DM
CKD prevention	GLP-1 RA reduce albuminuria and modify risk factors and reduction of a broad kidney composite outcome by 17%. Recommended in CKD.	No human trials yet. CVOT in T2DM suggest renoprotective effects with corrected for glycaemic control.

CVD prevention	GLP-1 RA reduce MACE in CVOT by 14% and a slightly lower risk of hospitalization for HF by 9%, reduction in all-cause mortality by 12%.	No direct trials yet. GLP-1 RAs for 7 days make a mild improvement of left ventricular function in patients with ST-segment elevation myocardial infarction (STEMI) as well as non-STEMI.

Dyslipidaemia, hypertension, and nonalcoholic fatty liver disease (NAFLD) prevention	No direct trials for GLP-1 RA on hypertension, NAFLD, and dyslipidaemia. CVOTs showed reduction of BP and dyslipidaemia, consistent with reduced all-cause mortality	Liraglutide vs. placebo in obese patients, BP, and fasting lipids lower with liraglutide. Liraglutide vs. placebo in obese patients, more frequent resolution of NAFLD, and less progression to fibrosis. Meta-analysis shows GLP-1 RA reduce ALT and improve NAFLD.

Prevention of PCOS, metabolic, and cardiovascular complications	GLP-1 RA improve insulin sensitivity and weight control, and therefore hyperandrogenism and metabolic complications in PCOS. Exenatide vs. metformin, exenatide induce remission of 56% of prediabetes in PCOS.	Open-label trial liraglutide, reduce weight. RCT liraglutide vs. metformin, liraglutide reduce BMI. RCT exenatide vs. metformin, exenatide lower BMI. Open-label trial exenatide, BMI -1 to -3.1. Exenatide vs. metformin, exenatide increase fertility, frequency and regularity of periods, and ovulatory rate.

Obesity & overweight prevention	GLP-1 RAs proven to induce weight loss and improve glycaemic control. Liraglutide body weight loss of 5.8% Semaglutide body weight loss of 6.9-9.6%	Pharmacotherapy for BMI > 30 or overweight with complications. RCT diet+exercise+/-liraglutide, found BMI -3+/-2.6 with liraglutide. RCT liraglutide vs. semaglutide vs. placebo, found 7.8% LOW with liraglutide, and 11-14% LOW with semaglutide.

Prevention of perioperative and postoperative complications of bariatric surgery	GLP-1 RA known to improve glycaemic control and treat the persistent/recurrent T2DM postbariatric surgery. No human trials yet investigating role of GLP-1 RA in success of bariatric surgery.	No human trials yet. GLP-1 rises after bariatric surgery and prevent the onset of T2DM postbariatric surgery.

LOW: loss of weight.

## Data Availability

Literature review using the following Medical Subject Heading (MeSH) terms including GLP-1 RA, guideline, safety, cardiovascular disease, chronic kidney diseases, obesity, dyslipidaemia, hypertension, nonalcoholic fatty liver diseases, polycystic ovarian syndrome, and perioperative complications of bariatric surgery has identified 4594 publications related to GLP-1 RAs' safety, guidelines in different countries, and potential preventive roles. These 4594 publications have been stored in research drive, password protected, and will be available on request from corresponding authors.
